# A Multi-Modal Face Recognition Method Using Complete Local Derivative Patterns and Depth Maps

**DOI:** 10.3390/s141019561

**Published:** 2014-10-20

**Authors:** Shouyi Yin, Xu Dai, Peng Ouyang, Leibo Liu, Shaojun Wei

**Affiliations:** Institute of Microelectronics, Tsinghua University, Beijing 100084, China; E-Mails: daixu@gmail.com (X.D.); oyangpeng12@163.com (P.O.); liulb@tsinghua.edu.cn (L.L.); wsj@tsinghua.edu.cn (S.W.)

**Keywords:** smart city, face recognition, multi-modal 2D + 3D, Complete Local Derivative Pattern, depth map, CLDP-Gabor, CLDP-Depth

## Abstract

In this paper, we propose a multi-modal 2D + 3D face recognition method for a smart city application based on a Wireless Sensor Network (WSN) and various kinds of sensors. Depth maps are exploited for the 3D face representation. As for feature extraction, we propose a new feature called Complete Local Derivative Pattern (CLDP). It adopts the idea of layering and has four layers. In the whole system, we apply CLDP separately on Gabor features extracted from a 2D image and depth map. Then, we obtain two features: CLDP-Gabor and CLDP-Depth. The two features weighted by the corresponding coefficients are combined together in the decision level to compute the total classification distance. At last, the probe face is assigned the identity with the smallest classification distance. Extensive experiments are conducted on three different databases. The results demonstrate the robustness and superiority of the new approach. The experimental results also prove that the proposed multi-modal 2D + 3D method is superior to other multi-modal ones and CLDP performs better than other Local Binary Pattern (LBP) based features.

## 1. Introduction

The emerging concept of the smart city has attracted considerable attention in the urban development policy field and academic research. It proposes that smart cities are defined by their innovation and their ability to solve problems and the use of Information Communication Technologies (ICTs) to improve this capacity [1]. A smart city can be regarded as a city-scale example of an Internet of Things (IoT) application. Wireless Sensor Networks (WSNs) and various kinds of sensors are the fundamental elements of the IoT.

One of the challenges the smart city faces is how to understand the data collected by the sensor network and make a decision. This is an application level topic mainly concerning pattern recognition and data mining based on the physical system. For example, like for smart surveillance, pedestrian detection, face recognition and behavioral analysis are exploited. In this paper, we mainly focus on the face recognition issue, which is extensively used in the smart video surveillance [2] and general identity verification (e.g., electoral registration, banking, and electronic commerce) [3].

Face recognition has been investigated intensively during the recent decades as a research focus of computer vision owing to its wide range of applications [4]. A lot of excellent achievements have been made, including: Fisherface [5], Gabor feature [6], Scale-Invariant Feature Transform (SIFT) features [7], the Principal Component Analysis (PCA) method [8], the Sparse Representation-based Classification (SRC) algorithm [9], *etc.* The performance of face recognition methods has increased dramatically. However, the robustness of face recognition still needs improvement. The results of most methods are more or less influenced by environmental changes, such as illumination variations, expression variations and pose variations. It is hard to find a method that can deal well with these variations.

As for 2D face recognition, various features are proposed. The recently proposed Local Binary Pattern (LBP) feature [10,11] which was originally designed for texture description, proves to be an effective local texture feature for face recognition. LBP shows robustness against pose and expression variations. It is also insensitive to monotonic gray-level variations caused by illumination variations. Owing to the robustness of LBP, various LBP-based face descriptors have been developed and proved successful in improving face recognition performance [12].

Recently 3D face recognition has become one of the hot topics in face recognition research [10]. The appearance of 3D sensors like Kinect have made the acquisition of depth data become cheaper and more convenient. Research related with depth map is becoming more and more popular. Obviously 3D face data can provide more geometric and shape information. Thus, it has inherent advantages when dealing with expression, pose and illumination variations. A series of methods for 3D face recognition have been proposed. They can be classified as 2D image-based, 3D image-based and multi-modal 2D + 3D systems [10].

In this paper, we propose a novel multi-modal 2D + 3D face recognition method, which takes full advantage of the metrics of both 2D and 3D images. A depth map is exploited for the 3D face representation. As for feature extraction, we propose a new feature model called Complete Local Derivative Pattern (CLDP). It adopts the idea of layering and has four layers: two local binary patterns with different radii, one high order derivative pattern, and one difference pattern. CLDP can provide more detailed description than the Local Binary Pattern or Local Derivative Pattern models alone. A depth map is regarded as an intensity image. In the whole system, we apply CLDP separately on Gabor features extracted from a 2D image and depth map. Then, we obtain two features: CLDP-Gabor and CLDP-Depth. The Whiten PCA (WPCA) algorithm is used on the two features to reduce the feature dimensionality and improve discriminative ability. The two features are combined together weighted by the corresponding coefficients in the decision level to compute the total classification distance. Since our feature has high discriminability, a simple classifier is enough to achieve high performance. Finally, the face recognition task is complemented by the nearest neighborhood classifier. The innovations of our work can be summarized in three points:

- We propose a novel multi-modal method which utilizes the metrics of both 2D and 3D images with the easily acquired depth map used for 3D representation.
- We present a discriminative feature model called CLDP which can provide more detailed descriptions than the Local Binary Pattern or Local Derivative Pattern alone.
- We apply LBP-based features to the depth map directly and prove the method is reasonable with analysis and experiment results.

The rest of paper is organized as follows: Section 2 introduces related work in detail. Section 3 introduces our method step by step. Experiments are presented in Section 4. Finally the conclusions are drawn in Section 5.

## 2. Related Work

As mentioned previously, various LBP-based features have been proposed to improve the face recognition performance. 3D face recognition is also a research focus. Whereas the featured CLDP model we propose is a kind of LBP-based feature and our face recognition is a multi-modal one, the two parts are introduced in details in this section.

### 2.1. LBP-Based Feature

In order to make the feature more discriminative, Huang *et al.* proposed a method called Extended Local Binary Pattern (ELBP) [13]. The ELBP descriptor not only performs binary coding by comparing the gray value of the central pixel with those of its neighbors, but also encodes their exact gray-value differences (GDs) using some additional binary units. Specifically, the ELBP feature consists of several LBP codes at multiple layers, which encode the GD between the central pixel and its neighboring pixels. The idea of layering is innovative and has also been adopted in [14].

LBP can be conceptually regarded as a non-directional first-order local pattern, which is the binary result of the original image. It has been proved that high-order descriptor can provide more detailed and more discriminative information. A series of high-order local descriptors have been proposed, such as Local Derivative Pattern (LDP) [15], Patterns of Oriented Edge Magnitudes (POEM) [15,16], *etc.* In the LDP method,(*n* − 1)*^th^* -order derivative images along four fixed directions are obtained first. Then the *n^th^* -order LDP is obtained in the way similar to the LBP by comparing the derivative value of central point with those of neighborhoods; For every pixel, the POEM feature is built by applying a self-similarity based structure on oriented magnitudes, calculated by accumulating a local histogram of gradient orientations over all pixels of image cells, centered on the considered pixel.

The proposed feature Local Tetra Pattern (LTrP) [17] encodes the relationship between the referenced pixel and its neighbors, based on the directions that are calculated using the first-order derivatives in vertical and horizontal directions. In [18], the Local Ternary Co-occurrence Pattern (LTCoP) encodes the co-occurrence of similar ternary edges which are calculated based on the gray values of center pixel and its surrounding neighbors. Our CLDP feature is also a kind of LBP-based feature. It takes advantage of the idea of layering and combines LBP and LDP together.

### 2.2. 3D Face Recognition

As mentioned previously, the 3D face recognition methods can be classified as 2D image-based, 3D image-based and multi-modal 2D + 3D systems [10]. The first class includes methods based on 2D images supported by some 3D data. The main idea is to utilize a general 3D model to improve the robustness. For example, Blanz *et al.* proposed a morphable face model to extract 3D shapes, textures and other 3D information parameters as features from single 2D images [19]. Recognition is accomplished by a classification algorithm using these features. It is important to propose a precise face model for this method, however, this turns out to be quite difficult.

The 3D image-based class refers to the methods that work on 3D facial representations, like range images or 3D polygonal meshes. Various methods have been proposed. The main purpose of the methods is to analyze facial surfaces and extrapolate shape information [20–22]. In [22] the Iterative Closest Normal Point (INCP) method is introduced for finding the corresponding points between a generic reference face and each input face. The proposed correspondence finding method samples a set of points for each face, denoted as the closest normal points. The INCP method achieves the state-of-art result on the Face Recognition Grand Challenge database. However, it is only robust to expression variations.

The methods combining 2D image and 3D image information belong to the last category. The method proposed by Chang *et al.*, executes the PCA algorithm on the intensity and range images, respectively, and then combines the two results when computing the classification distance [23]. The experiment results prove that the method combining 2D and 3D results with their corresponding weights outperforms the method that only uses either 2D or 3D results alone.

Depth proved to provide more robust face representations than intensity [24] and was exploited for face recognition in several methods [23,25,26]. A depth map is regarded as a grey-scale image and the PCA algorithm is utilized. In [23,26], the methods combine depth information with 2D intensity images. Nanni and Lumini proposed an improved boosting algorithm, named RegionBoost, and described its application in developing a fast and robust invariant Local Binary Pattern histogram-based face recognition system [27]. They made more efforts on the classification algorithm. In [28], a systematic framework was proposed. It can fuse 2D and 3D face recognition at both the feature and decision levels, by exploring synergies between the two modalities at these levels. However the feature they used is still simple LBP.

The Multi-modal Sparse Coding (MSC) method is presented in [29]. The canonical depth map and texture of a query face are sparse approximated from separate dictionaries learned from training data. The texture is transformed from the RGB (Red Green Blue color space) to Discriminant Color Space before sparse coding and the reconstruction errors from the two sparse coding steps are added for individual identities in the dictionary. These multi-modal methods inspired us to propose our method.

## 3. Methodology

**Figure 1.**
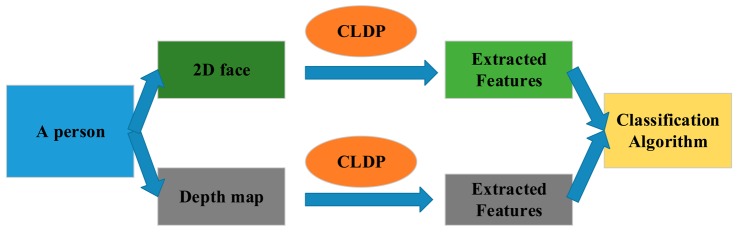
The framework of the whole system.

In this section, the whole method is described in details. The framework is shown in Figure 1. In Section 3.1, we propose our feature Complete Local Derivative Pattern model which contains both LBP and LDP at the same time. Then the model is applied to the 2D image and depth map respectively. For 2D images, we introduce the Gabor feature in Section 3.2. The CLDP is applied on the Gabor features which are extracted from the 2D image. As for the depth map, it is regarded as the intensity image. The CLDP is applied on the depth map directly in Section 3.3. Then, the WPCA algorithm is implemented on the two kinds of features. Finally the two kinds of features are combined together weighted by the corresponding coefficients. The recognition is finally completed by the classification algorithm. The whole system is described again in Section 3.4.

### 3.1. Complete Local Derivative Pattern

The CLDP consists of three steps. The binary coding and label coding are similar to LBP. It utilizes difference and high order (to be exact, 2nd-order) derivative information. CLDP takes advantage of layering. Two layers of LBP, one layer of LDP and one layer of difference are included in the CLDP. LBP and the difference can be regarded as the 1st-order derivative pattern, so the feature model is called Complete Local Derivative Pattern. Like with other LBP-based features, a spatial histogram is chosen for the feature representation.

#### 3.1.1. Binary Coding (Difference & High Order Derivative)

**Figure 2.**
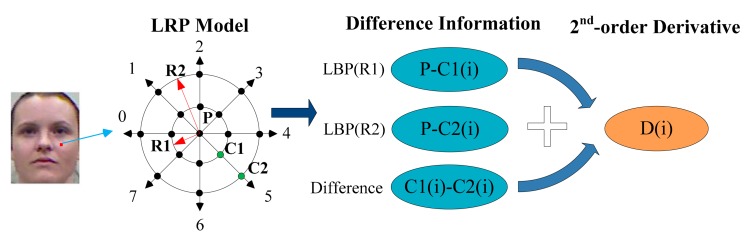
The feature decomposition operation.

For each pixel *P*, a Local double Radii Pattern (LRP) model is applied as shown in Figure 2. LRP is a double LBP with different radii centered in the same point. We choose eight specific directions (0°, 45°, 90°, 135°, 180°, 225°, 270°, 315°) based on LRP for better discrimination. There are three pixels *P,C*2(*i*),*C*1(*i*) forming relationships at each orientation, and here *i* means the direction number. The difference between each two is used for the 1st-order derivative information, which is the basic and original information. On the other hand, the 2nd-order derivative *D*(*i*) reveals the change trends between data. This step is shown in Figure 2. The difference values are transformed to codes 0 or 1, which is similar to LBP. The binary coding function is formulated as below:

(1) $$BinaryCoding(value)=\{\begin{array}{cc} 1 & \left(value>0\right)\\ 0 & \left(else\right) \end{array}$$

As for the 2nd-order derivative part, we do not calculate the 2nd-order derivative value. The signs of the difference values are used to transform the 2nd-order derivative information to binary code, which is similar to LDP. The function is formulated as below:

(2) $$D(i)=\{\begin{array}{cc} 1 & [P-C1(i)]\cdot [C1(i)-C2(i)]>0\\ 0 & [P-C1(i)]\cdot [C1(i)-C2(i)]\leq 0 \end{array}$$

#### 3.1.2. Layering & Label Coding

**Figure 3.**
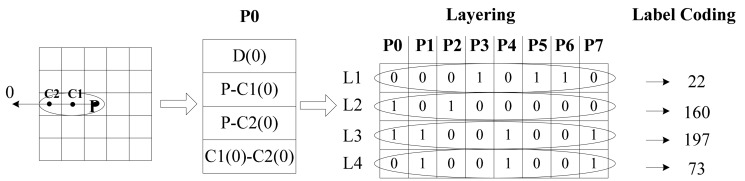
The layering and label coding step.

We can obtain 32 data items for one point which is too sparse to process in the cascade mode. Then we creatively bring in the layer method. The encoded features are listed in turn as shown in Figure 3. For computational convenience, we transform the eight binary codes at each layer into a decimal number. It is easy to know that L1 is the 2nd-order Local Derivative Pattern, L2 and L3 are the Local Binary Patterns and L4 is the extra difference information. These four decimal numbers contain almost all the required information. Besides, intrinsic and latent relations between data are exposed.

**Figure 4.**
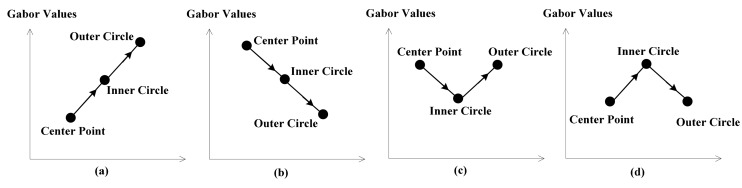
Different situations with the same D value.

Figure 4 below will show why CLDP is superior to LDP and we will analyze the meaning of L2, L3 and L4. High order derivative proves to be more discriminative, however, sometimes it is fuzzy. As shown in Figure 4, (a) and (b) have the same LDP value 1, while (c) and (d) have the same LDP value 0. Although their L1 values are equal, they in fact represent adverse trends. That’s why L2 and L4 are needed. The combination of L2 and L4 precisely overcomes this defect and assists L1 in describing the changing trend. L3 can provide another layer of LBP in a different radius. The Local Derivative Pattern is extended to be complete with L1, L2, L3, and L4. Which makes CLDP superior to LDP.

#### 3.1.3. Histogram

A spatial histogram is adopted to represent features. Label values of all the pixels in one layer make up a labeled image. Four labeled images are obtained after label coding. We first divide the labeled images into several blocks, then extract a local histogram for every individual block and finally concatenate all the histograms of different blocks and directions into a histogram which is the ultimate characteristic representation of the original image. Finally four histograms are concatenated to a complete histogram. The dimension of the histogram is determined by the number of blocks and bins. Histograms are commonly used in feature extraction. They are not only a good way to greatly reduce the data in the labeled image, but also a more reliable and robust way to represent the features.

### 3.2. CLDP-Gabor

The Gabor feature is another excellent texture feature intensively used in face recognition [6,30,31]. Daugman discovered that simple cells in the visual cortex of mammalian brains can be modeled by Gabor functions [32]. Thus, image analysis by the Gabor functions is similar to perception in the human visual system. It was proved in [15,33,34] that Gabor-based and LBP-based features are complementary to each other. LBP features can extract the local texture details, whereas Gabor features can extract texture information on a broader range of scales. The most used way of combination is by applying Gabor filters to original image and, then, LBP to the processed image. In LDP [15], Zhang *et al*. also proved that after extending LDP to Gabor feature images the performance of the LDP feature increased. The new feature is called GLDP.

For the 2D image, we apply CLDP on the Gabor feature to enhance the object representation capability. The new feature is called CLDP-Gabor feature, CLDP-G for short. The Gabor feature is extracted from an image by convolving the raw image with a set of Gabor filters at different frequencies and orientations. The Gabor filters (kernels) can be formulated as follows [6]:

(3) $$\psi_{u,v}(z)=\frac{{∥k_{u,v}∥}^{2}}{\sigma^{2}}e^{\left(-{∥k_{u,v}∥}^{2}{∥z∥}^{2}/2\sigma^{2}\right)}\left[e^{ik_{u,v}z}-e^{-\sigma^{2}/2}\right]$$

where $z=\left(\begin{array}{c} x\\ y \end{array}\right)$, $k_{u,v}=\left(\begin{array}{c} k_{jx}\\ k_{jy} \end{array}\right)=\left(\begin{array}{c} k_{v}\cos\phi_{u}\\ k_{v}\sin\phi_{u} \end{array}\right)$, *k_v_* =*π*/2^(^*^v^*^+2)/2^, *ϕ_u_* = *u*(*π*/8), *v* = 0,…,*v*_max_ −1, *u* = 0,…, *u*_max_ −1, *v* is the frequency, *u* is the orientation. We set *v*_max_ = 5,*u*_max_ = 8, *σ* = 2*π* in our work which is the same with most other related works [6,15]. Let *I*(*x*, *y*) denote the gray level distribution of an image. The Gabor wavelet representation of an image is defined as follows:

(4) $$G_{u,\text{v}}(z)=I(z)*\psi_{u,v}(z)$$

where *z* = (*x*, *y*) , * denotes the convolution operator, and *G_u,v_* (*z*) is the convolution result correspon-ding to the Gabor kernel at orientation *u* and scale *v*. Therefore, the set *S* = {*G_u,z_* (*z*):*u* ∈ {0,…,7}, *v* ∈ {0,…,4} } forms the Gabor wavelet representation of the image *I*(*z*). The real and imaginary parts of *G_u,v_* (*z*) are denoted as Re(*G_u,v_*(*z*)) and Im(*G_u,v_*(*z*)) respectively. CLDP is applied on Re(G*_u,v_*(*z*)) and Im(*G_u,v_*(*z*)) of each representation. Two histograms are obtained. The histogram of CLDP-G (HCLDP-G) is defined as:

(5) HCLDP-G={HCLDP-Re(Gu,v(z)),HCLDP-Im(Gu,v(z))∣u=0,…7;v=0,…4}

where HCLDP-Re(G*_u,v_*(*z*)) and HCLDP Im(G*_u,v_*(*z*)) are the histograms extracted from the real parts and imaginary parts of *G_u,v_* (*z*) respectively. All histograms are concatenated to a whole one, as the final feature representation of CLDP-G.

### 3.3. The Use of Depth Information

**Figure 5.**
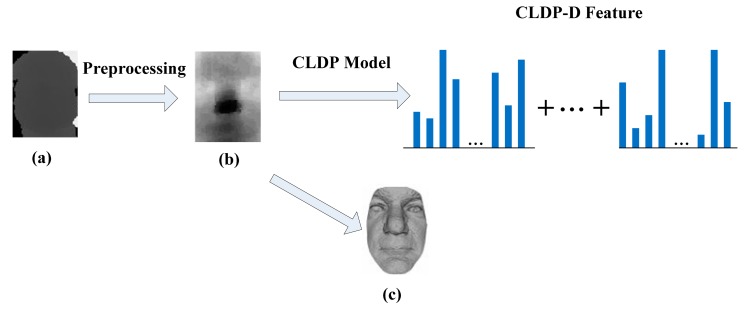
The process of extracting CLDP-D feature. (**a**) A depth map displayed as an intensity image; (**b**) A depth map after preprocessing displayed as an intensity image; (**c**) 3D face rendered as a smooth shaded surface.

As mentioned previously, the depth map is more robust and discriminative. Depth-related features are becoming more and more popular with the acquisition of depth maps becoming easier. We can think of a depth map as a grey-scale image. The value of each pixel on a grey-scale image represents the intensity of this point, while the value of each pixel on a depth map denotes the distance between the camera and the surface point. The distance can be regarded as a kind of variable which is analogous to the intensity. Then, the depth map is analogous to the grey-scale image. Figure 5 shows a depth map displayed as a grey-scale image. The brightest pixel represents the surface point that is nearest to the camera. Depth map reflects the geometric and shape information of the face.

The depth map obtained by Kinect is of low resolution. There are spikes and holes in the range data. Before we use the data, some preprocessing is conducted such as Gaussian smoothing, median filtering and hole filling. The depth map after preprocessing is shown in Figure 5b. The depth map can be rendered as a smooth shaded surface as Figure 5c shows. However, we use the raw depth map as a 2D intensity image and extract the features in another way.

In [23,26], a depth map is regarded as a grey-scale image. They separately execute the PCA algorithm on intensity images and depth maps. Then the two parts are combined together. In our work, we apply CLDP on the depth map and obtain the CLDP-Depth feature, CLDP-D for short, as shown in Figure 5. As mentioned above, the depth can be analogous to the intensity and the depth map is analogous to the grey-scale image. The LDP feature is insensitive to monotonic gray-level variations caused by illumination variations, because it can describe the variation trend. Analogously, CLDP-G is insensitive to monotonic depth variations caused by pose and expression variations. On the other hand, illumination variations have little effects on depth image, so the feature is also intensive to illumination variations. So our method of applying CLDP directly on depth map is reasonable.

### 3.4. Face Recognition Method

**Figure 6.**
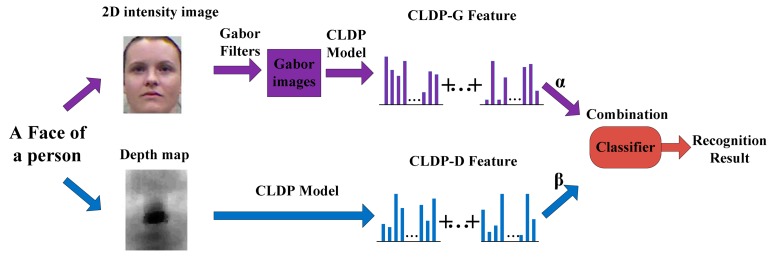
The whole framework of our multi-modal 2D + 3D face recognition method.

The entire face recognition method is shown in Figure 6. We acquire an intensity image and a depth map at the same time for each face. Then the novel feature model CLDP is applied on the two parts separately. Two kinds of features: CLDP-G and CLDP-D are extracted. With respect to combining them together, we design a form of weights, which proves to be efficient in [23]. The distance metrics calculated using CLDP-G and CLDP-D features are denoted by *ϕ*_G_ and *ϕ*_D_ separately. The total distance metric is formulated as below:

(6) $$\varphi =\alpha \cdot \varphi_{G}+\beta \cdot \varphi_{D}$$

where *α* denotes the weight of CLDP-G and *β* denotes the weight of CLDP-D, *α, β* ∈ [0,1], *α* + *β* = 1. Finally the face can be recognized correctly by the classifier. The nearest neighbor classifier is chosen as the classification algorithm. Because our feature is designed quite elaborately, a simple classifier is enough to achieve excellent recognition results.

It is necessary to point out that we apply the Whitened Principal Component Analysis (WPCA) on CLDP-G and CLDP-D separately before classification. WPCA has been proved to be an effective approach to reduce feature dimensionality and improve discriminative ability. After feature extraction, the feature matrix *F* is obtained. Exploiting the PCA method we can get the PCA matrix *P* which is made up of the eigenvectors of *F*. The corresponding eigenvalues are {*λ*_1_, *λ*_2_… *λ_i_*…}. Then the feature matrix is processed by WPCA as below:

(7) $$F_{WPCA}=\Lambda^{-\frac{1}{2}}PF$$

where $\Lambda^{-\frac{1}{2}}=diag\{{\lambda_{1}}^{-\frac{1}{2}},{\lambda_{2}}^{-\frac{1}{2}}\ldots {\lambda_{i}}^{-\frac{1}{2}}\ldots \}$.

## 4. Experimental Section

In this section, intensive experiments are conducted to evaluate the performance of the proposed method. Our approach is compared with other methods on the same databases. For classification, histogram intersection is adopted as the similarity measure, because it proves to be a good choice for histogram matching [11]. The nearest neighborhood is exploited for final classification.

Three groups of experiments are performed in this section. The first experiment is conducted on the Facial Recognition Technology (FERET) database [35] to evaluate the performance of our CLDP feature model. The second and the third experiments are conducted separately on CurtinFaces database [36,37] and Notre-Dame Dataset collection D [23,38] to compare our method with several other methods. The abbreviations of methods used in the experiments are given in the Table 1.

### 4.1. Experiment on the FERET Database

#### 4.1.1. Database and Experiment Settings

**Figure 7.**
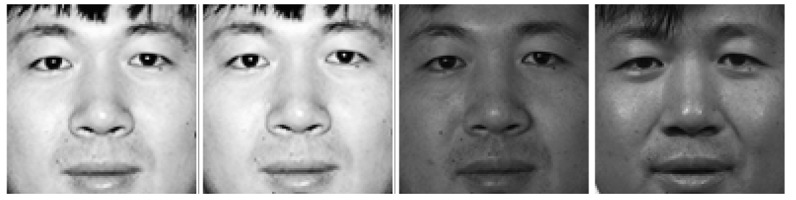
The faces of one subject from fa, fb, fc and dupI subsets (left to right) in turn.

The FERET database consists of five subsets. Subset *fa*, including 1196 images of 1196 subjects, is set as gallery set. The other four subsets are used as probe sets: *fb* (containing expression variations, 1195 images of 1196 subjects), *fc* (containing illumination variations, 194 images of 194 subjects), *dupI* (taken later in time between one minute to 1031 days, 722 images of 243 subjects), *dupII* (taken at least 18 months later, 234 images of 75 subjects). The faces of one subject is shown in Figure 7. All images are properly aligned, cropped and resized to 128 × 128 with the centers of the eyes fixed at same horizontal line. No further preprocessing is performed. LBP, LDP [15], POEM [39], LTrP [17], LTCoP [18] and our CLDP are tested on the database and nearest neighbor classifier is chosen as the classification algorithm. The blocks of the image are set as 12 × 12.

#### 4.1.2. Experiment Results and Analyses

FERET database contains expression, illumination and aging variations. The experiment on this database evaluates the robustness of features against these variations. As shown in Table 2, our CLDP achieves the best recognition rates on all the subsets. The results on *fb* and *fc* are quite high even with simple LBP. This is owing to that LBP is inherently intensive to expression and illumination variations. The *dupI* and *dupII* seem much more difficult to deal with. Only POEM and our method obtain excellent results. The methods except LBP all contain the derivative information and the improvement shows that the derivative information is more discriminative. LTrP and LTCoP which are proposed for image retrieval still achieve good results. However, the recognition rates on *dupI* and *dupII* are much lower than with our method.

### 4.2. Experiment on the CurtinFaces Database

#### 4.2.1. Database and Experiment Settings

We use the online CurtinFaces database in this experiment. The data is captured using a Kinect Sensor. Unlike traditional 3D scanners, Kinect trades data quality for low cost and high speed, which is usually a requirement for broad practical applications. In our experiments, we use the all 97 images of each subject, including males and females. The database consists of 5044 images and 52 individuals with variations in poses (*P*), illumination (*I*), facial expressions (*E*) and disguise (*D*) simulating a real-world uncontrolled face recognition problem. The 3D point cloud and RGB data are stored in Matlab (.mat) format as XYZRGB (XYZ denotes the three-dimensional coordinate (*x,y,z*) of the point and RGB denotes the values of the three channels: red, green and blue for the same point). To study the robustness of different changes, we divide sample images into three new subsets according to their characteristic as shown in Table 3. Curtin-PE consists of 7 kinds of pose variations × 7 kinds of expression variations and first three faces of each subject. Curtin-IE consists of 5 kinds of illumination variations × 7 kinds of expression variations. Curtin-D consists of faces disguised with sunglasses or hands under different poses or illumination conditions. All images are properly aligned, cropped and resized to 90 × 120 with the centers of the eyes fixed at same horizontal line.

**Figure 8.**
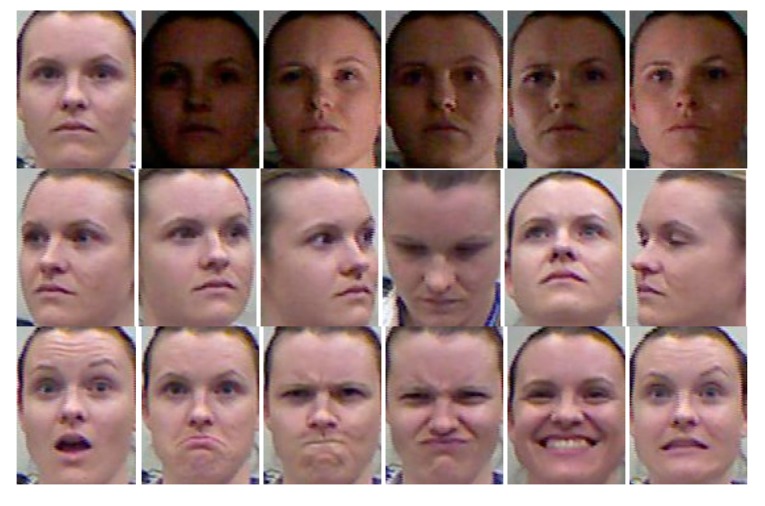
The chosen 18 images of person 01 for gallery data.

The experiment method is the same as shown in Figure 6. In the following experiments we select 18 images per subject (see Figure 8) for gallery data. Each gallery image contains only one of the three variations (illumination, pose and expression). The rest images of the three subsets are used for test data. We take CLDP-G, CLDP-D, LDP-GD and LBP-GD methods for horizontal comparison as they are the basics of our feature. And take MSC and INCP for vertical comparison.

#### 4.2.2. Weights Definition

**Figure 9.**
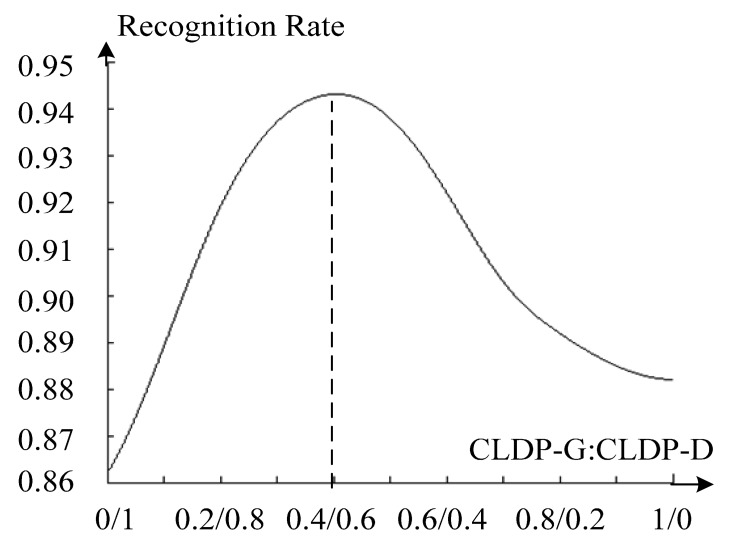
Results of changing the weights of CLDP-G and CLDP-D.

To decide the weights *α* and *β*, we make an experiment with *α* ranging from 0 to 1 (*β* = 1 − *α*)). The average recognition rate of the total database is presented in Figure 9 below. It shows the recognition rate reaches the maximum value when *α* : *β* is equal to 0.4:0.6. We can also draw a conclusion that the two features are complementary to each other and perform much better together. As to why the extreme point is 0.4:0.6, we account this for that depth information seems a little more stable and robust than 2D features overall.

In the following experiments we set the *α* : *β* as 0.4:0.6. Recognition rate is the most representative metric to measure recognition performance. It illustrates the accuracy of methods which is our final goal. Besides, we introduced a new metric—Receiver Operating Characteristic (ROC) curve to state the robustness of methods. The smaller the area below the curve is, the more robust the method is.

#### 4.2.3. Experiment Results and Analyses

**Figure 10.**
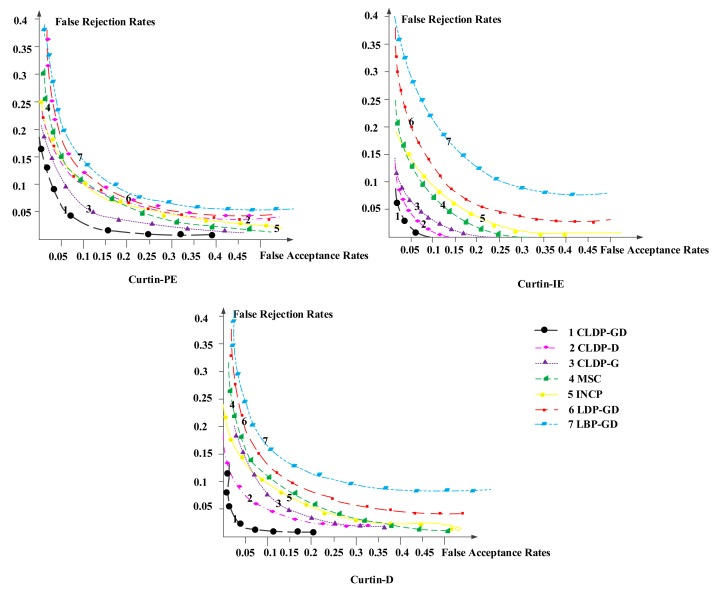
The ROC curves of corresponding methods on the three subsets.

The recognition results are shown in Table 4. We can see no matter on which database, our method obtains the best recognition rate, which is much higher than that achieved with other methods. The corresponding Receiver Operating Characteristic (ROC) curves are shown in Figure 10. The curves of our method are always in the bottom, far below other methods and appear milder. Comparing results on those three subsets, the average recognition result on Curtin-IE is the best, followed in turn by Curtin-PE, Curtin-D. Detailed analyses are shown in the following sections.

**Figure 11.**
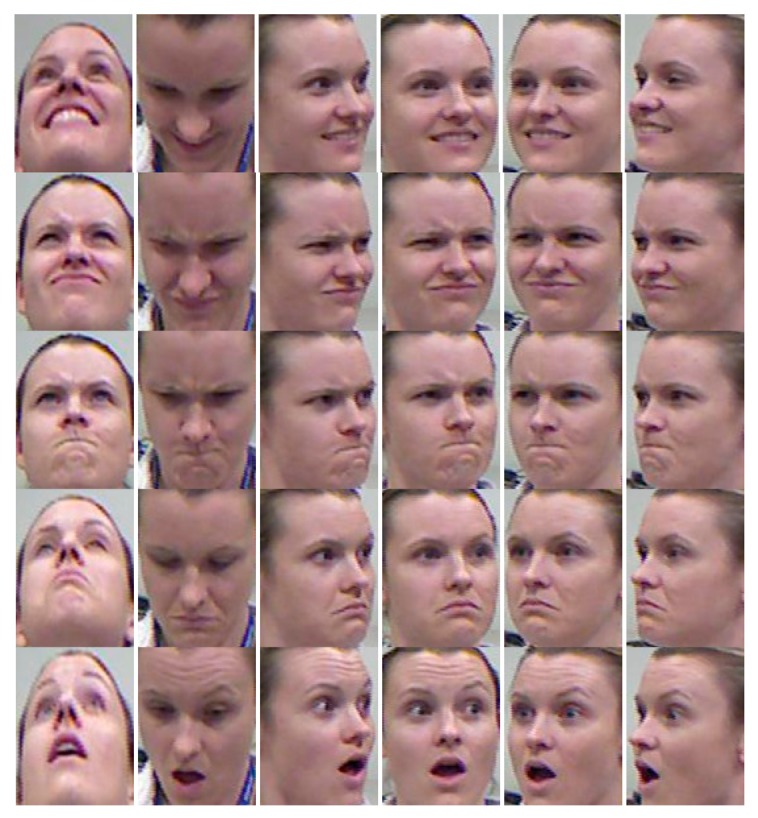
Sample of testing images in the Curtin-PE subset.

The sample images of Curtin-PE are shown in Figure 11. Curtin-PE primarily changes pose and expression simultaneously which seems a tough task to handle. The results on the Curtin-PE subset show that no method can get recognition rate higher than 95%. Even the MSC and INCP methods cannot deal with them well. However, our method can obtain 93.7% recognition rate. This power validates the advantages of CLDP-GD in that it can handle pose variations and expression variations.

When we compare CLDP-G (91.8%), CLDP-D (89.9%) with CLDP-GD, we can find the CLDP-G method obtains higher recognition than CLDP-D in this case. This may because depth feature changes greatly with pose changes, while texture features are little influenced. Texture featurew reflect facial information and CLDP-G feature especially shows the facial contour that even a side face with large expression variations can lead to accurate recognition. In spite of this, the combination of CLDP-G and CLDP-D achieves better result than either one of them, which demonstrates the robustness of our method.

The CLDP-GD method achieves a considerable improvement of recognition rate compared with the LBP-GD (82.3%) and LDP-GD (85.4%) method, which validates the superiority of the CLDP feature model. In addition, the LDP-GD method also achieves a nice result. This indicates that dynamic changing trend is effective for face recognition under pose and expression variation conditions.

**Figure 12.**
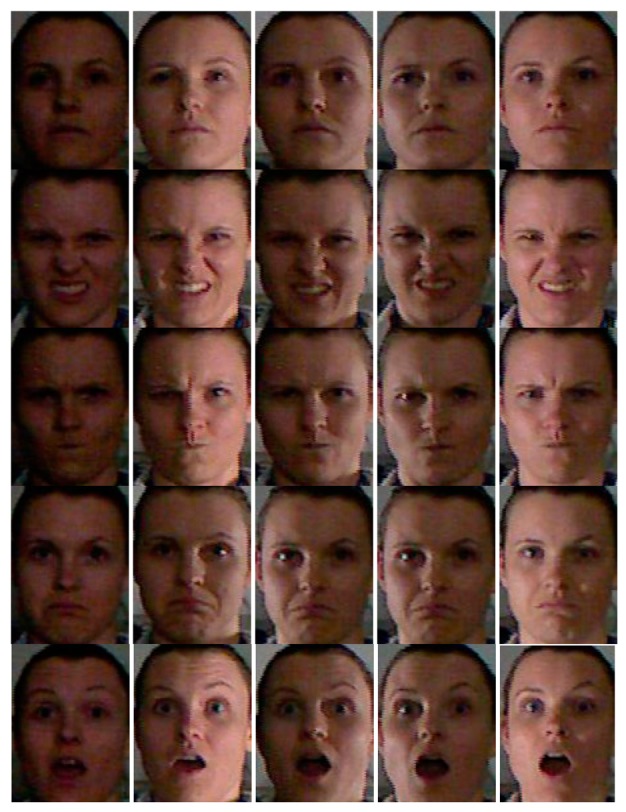
Sample of testing images in the Curtin-IE subset.

The sample images of Curtin-IE are shown in Figure 12. The Curtin-I database mainly changes the illumination and expression simultaneously, which is a little simpler than the Curtin-PE one. Although the six methods all perform well expect LBP-GD, the differences between our method and others are still distinct. We get the highest improvement of 13.9% on this database: 96.8% for our method and 82.9% for LBP-GD method. It should be noted that CLDP-D method obtains a similar performance as our method. We attribute this to the fact that illumination has no effect on depth and expression variations have little effect on CLDP-D features. To some extent, expression variations may disturb the texture feature, but the changes are eliminated after we extract the change trends with the CLDP model. This can also be seen when we compare the CLDP-GD results with the LBP-DG and LDP-GD method results.

**Figure 13.**
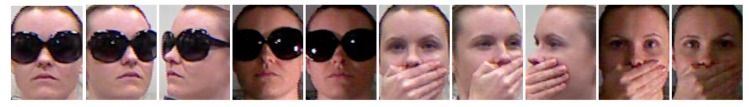
Sample of testing images in the Curtin-D subset.

The sample images of Curtin-D are shown in Figure 13. The faces in the Curtin-D database are disguised with sunglasses or hands under illumination or pose variations. It is the most complicated one of the three subsets. No method performs well. Though our method gets the best performance, the recognition rate is still below 90% (88.6%). We think this is mainly because the Gabor feature and LBP-based features are inherently not insensitive to disguise. What is notable is that the CLDP-D method achieves a fine result (87.1%) and contributes much more to the multi-modal method than CLDP-G. To solve the disguise problem better, we propose to choose a sparse representation-based classification algorithm for classification which will be studied comprehensively in our future work.

Throughout the total results, for horizontal comparison (with MSC and INCP), our method shows on average a 4.9 percent improvement. As for longitudinal comparison (with CLDP-G, CLDP-D, LBP-GD and LDP-GD), the gains based on these are even larger.

### 4.3. Experiment on the Notre-Dame Dataset Collection D

#### 4.3.1. Database and Experiment Settings

**Figure 14.**
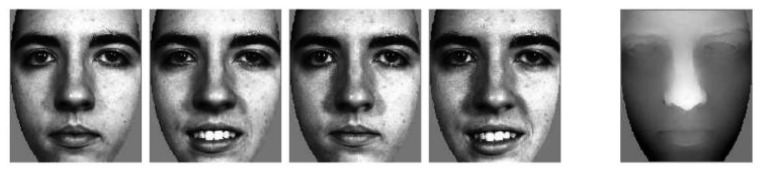
Four 2D images (left to right: FALM, FBLM, FALF, and FBLF) and one 3D image (rightmost) of a person acquired in each session.

This experiment is conducted on the Notre-Dame Dataset collection D. It consists of a total of 198 different persons who participated in two or more sessions of usable data. Two four-week sessions were conducted for data collection, with approximately a six weeks’ time lapse between the two. The 3D range images were acquired using a Minolta Vivid 900 (Konica Minolta, Tokyo, Japan) which is much more accurate than the Kinect. A 640 × 480 range image is produced by the 3D scanner and images of dimension 1704 × 2272 are produced by the 2D camera. Each 3D image is corresponding with 4 2D images. The 3D image is under one central spotlight and normal facial expression. As for the 2D images, each subject was asked to have one normal expression (FA) and one smile expression (FB), once with three spotlights (LM) and a second time with two side spotlights (LF). One sample of 2D and 3D images of one person acquired in a session is shown in Figure 14 below.

We take the 3D image and 2D image under FALM conditions of each subject in the all different sessions as the gallery data. The remaining pairs of 2D and 3D images are used as test data. The two kinds of representations have both been resized to the same 120 × 100 dimensions before recognition. CLDP-GD, CLDP-G, CLDP-D, INCP, M-PCA, RegionBoost, LtF, MSC are compared on the database. LBP-GD, LDP-GD and other LBP feature-based methods are not included in this experiment because the experiments on the FERET database and the CurtinFaces database have already proven that our CLDP method is superior to them.

#### 4.3.2. Experiment Results and Analyses

**Figure 15.**
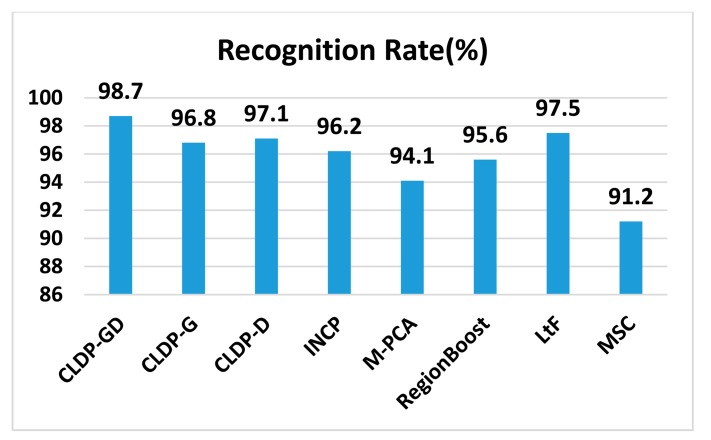
The experimental results on the Notre-Dame dataset.

The recognition results are shown in Figure 15. This dataset seems not so tough to deal with, because all methods achieve recognition rates above 91%. It should be pointed out that our CLDP-GD approach achieves the best recognition rate (98.7%). The CLDP-G method that only uses 2D data and the CLDP-D method that only uses 3D data both obtain quite excellent results, but the multi-modal method performs better. The improvements are 1.9% and 1.6%, respectively. The CLDP-GD, M-PCA, RegionBoost, LtF, MSC are all multi-modal 2D + 3D methods. Our CLDP-GD outperforms the other multi-modal ones; we owe this to the superiority of our CLDP feature model. The feature of M-PCA is quite simple eigenfaces. As for RegionBoost and LtF, the feature used is only LBP. Though RegionBoost uses the boosting algorithm and LtF fuses 2D and 3D features at both feature and decision level, the results are not as good as ours. As for MSC, it can handle well the variations in disguise which are not involved in this dataset.

## 5. Conclusions

In this paper, we have presented a new face recognition method integrating 2D texture and 3D depth information for smart city applications based on WSNs and various kinds of sensors. We have proposed a novel feature data processing model called CLDP. The model decomposes data into four layers: two local binary patterns with different radii, one high order derivative pattern, and one difference pattern. After applying CLDP separately on the 2D intensity image part and the 3D depth map, we obtain two features: CLDP-Gabor and CLDP-Depth. The two parts are finally combined together, weighted by their corresponding coefficients, for recognition. We conducted extensive experiments on three databases. The highest recognition rates and nice ROC curves show our method is robust and can tremendously improve the quality of face recognition. The experimental results also prove that our multi-modal 2D + 3D method is superior to other multi-modal ones and CLDP is superior to other LBP-based features. With the emergence of low cost 3D sensors (e.g., Kinect), our approach will show more practical value for the realization of the smart city. In the future, a sparse representation-based classification model will be studied and integrated with our multi-modal method to improve the recognition rate, especially when dealing with disguised subjects.

## Figures and Tables

**Table 1.** The abbreviations of methods and corresponding description.

**Method**	**Description**
CLDP-GD	The method we proposed that exploits both CLDP-Gabor feature and CLDP-Depth feature.
CLDP-G	The method that only utilizes CLDP-Gabor feature and 2D intensity image.
CLDP-D	The method that only utilizes CLDP-Depth feature and depth map.
LDP-GD	The method that first extracts Gabor features from the 2D intensity image of a face, then applies LDP feature model to Gabor images and depth map separately, finally combines the two parts together.
LBP-GD	The method similar to CLDP-GD and LDP-GD with LBP feature.
INCP	The 3D method proposed in [22].
M-PCA	The multi-modal PCA method proposed in [23].
RegionBoost	The multi-modal method proposed in [27].
LtF	The multi-modal method proposed in [28].
MSC	The multi-modal method proposed in [29].

**Table 2.** The recognition rates on the FERET database.

**Method**	**Recognition Rate (%)**			

	***fb***	***fc***	***dupI***	***dupII***
LBP	87.2	74.43	63.71	56.84
LDP (order = 3)	86.03	95.33	75.83	70.59
LTrP (order = 3)	88.16	94.37	72.53	71.58
LTcoP (order = 3)	92.47	97.94	80.36	78.39
POEM	96.15	98.45	90.12	87.78
CLDP	97.82	100	93.28	90.91

**Table 3.** Introduction of the three new subsets.

**Person ID-Image ID**	**Introductions**	**Subsets**
xx-01∼xx-52	Pose and expression variations simultaneously	Curtin-PE
xx-53∼xx-87	Illumination and expression variations simultaneously	Curtin-IE
xx-88∼xx-97	Disguise under different poses or illumination conditions	Curtin-D

**Table 4.** The recognition rates on the CurtinFaces database.

**Method**	**Curtin-PE**	**Curtin-IE**	**Curtin-D**
CLDP-GD	93.7%	96.8%	88.6%
CLDP-D	91.8%	94.4%	87.1%
CLDP-G	89.9%	92.5%	83.1%
MSC	87.1%	91.6%	85.5%
INCP	88.0%	93.4%	83.2%
LDP-GD	85.4%	88.5%	79.5%
LBP-GD	82.3%	82.9%	76.1%
